# Analysis of *BRCA1*- and *BRCA2*-Related Pancreatic Cancer and Survival

**DOI:** 10.1001/jamanetworkopen.2023.45013

**Published:** 2023-11-27

**Authors:** Ben Boursi, E. Paul Wileyto, Ronac Mamtani, Susan M. Domchek, Talia Golan, Ryan Hood, Kim A. Reiss

**Affiliations:** 1 Department of Oncology, Sheba Medical Center, Tel HaShomer, Israel; 2 Center for Clinical Epidemiology and Biostatistics, University of Pennsylvania, Philadelphia; 3 Abramson Cancer Center, University of Pennsylvania, Philadelphia

## Abstract

This cohort study compares the outcomes of patients with *BRCA1* and *BRCA*-related pancreatic cancers using 2 large data sets.

## Introduction

Patients with pancreatic cancer (PDAC) and pathogenic variants (PVs) in *BRCA1* or *BRCA2* have historically been grouped together.^1,2^ However, preclinical and clinical data in other *BRCA*-related tumors suggests that the biological implications of *BRCA1* and *BRCA2* PVs may be different.^3,4,5^ To explore this further, we compared outcomes of patients with *BRCA1* vs *BRCA2*-related PDAC using 2 large data sets.

## Methods

The institutional review board at the University of Pennsylvania deemed this study exempt and waived informed consent because deidentified data was used. We followed the Strengthening the Reporting of Observational Studies in Epidemiology (STROBE) reporting guideline.

Our cohort was derived from the national Flatiron Health database and from a local University of Pennsylvania database. The study population consisted of adult patients with germline PVs in *BRCA1* or *BRCA2* and a new diagnosis of PDAC between 2005 to 2021. Only individuals with complete data, including age, sex, ECOG status at diagnosis, resection status, and treatment information were included in the analysis. The primary outcome was overall survival (OS), calculated as the time of diagnosis to date of death or last follow up.

Adjusted hazard ratios (aHRs) and 95% CIs were calculated for comparison of OS between patients with *BRCA1* PVs vs *BRCA2* PVs using the Cox-proportional-hazards model. Kaplan-Meier curves compared OS between *BRCA1* vs *BRCA2* PVs, stratified by resection status and use of platinum-based therapies. *P* ≤ .05 was considered statistically significant. All reported *P* values are 2-sided. Analyses were performed using Stata version 16 (StataCorp). Data were analyzed from June 2022 to June 2023.

## Results

This study included 234 consecutively identified patients with germline *BRCA* PVs and a diagnosis of PDAC (165 [70.5%] *BRCA2*; 69 [29.5%] *BRCA1*). Of patients with *BRCA1*, 33 of 69 (47.8%) were males with a median (IQR) age of 63 (55-70) years. Of patients with *BRCA2*, 84 of 165 (50.9%) were males, and the median (IQR) age was 64 (58-70) years. There were no significant differences in age, sex, performance status, year of diagnosis, or rates of exposure to platinum or poly (ADP-ribose) polymerase (PARP) inhibitor therapy between groups. Patients with *BRCA2* PVs were more likely to undergo primary tumor resection (50 [30.3%] vs 15 [21.7%]).

Survival outcomes by *BRCA* status, stratified by stage and platinum exposure, are shown in the Table and Figure. In the total cohort, patients with *BRCA2* PV had improved OS compared with patients with *BRCA1* (mean [SE], 28.98 [2.53] months vs 23.36 [1.55] months), corresponding to an aHR of 0.64 (95% CI, 0.44-0.94; *P* = .02). In patients who underwent resection (Figure), *BRCA2* PV was associated with improved OS compared with *BRCA1* PVs (mean [SE] months, 55.59 [11.95] vs 24.54 [2.70] months; aHR 0.30 [95% CI, 0.13-0.70]; *P* = .006); in patients not undergoing resection, there were no appreciable differences in survival by *BRCA* status (24.47 months vs 21.42 months; aHR 0.67; 95% CI, 0.42-1.07). In patients who were exposed to platinum (Figure), *BRCA2* PVs were associated with improved survival compared with *BRCA1* PVs (mean [SE] OS, 32.26 [7.62] months vs 23.36 [2.89] months; aHR, 0.59 [95% CI, 0.38-0.93]; *P* = .02). When neither group received platinum, the survival advantage associated with *BRCA2* PVs decreased (mean [SE] OS, 24.84 [3.52] months vs 21.42 [5.39] months; aHR 0.44 [95% CI, 0.21-0.92]; *P* = .03).

**Table.** Patient Outcomes According to *BRCA* Status and Stratified by Resection Status and Platinum Exposure

Patient Outcomes	HR (95% CI)		Median OS (SE), mo
	Unadjusted	Adjusted	
**Resected (early stage) patients**			
*BRCA2* variant	0.45 (0.22-0.94)	0.30 (0.13-0.70)	55.59 (11.95)
*BRCA1* variant	1 [Reference]	1 [Reference]	24.54 (2.70)
**Unresected (locally advanced or metastatic) patients**			
*BRCA2* variant	0.75 (0.49-1.15)	0.67 (0.42-1.07)	24.48 (2.50)
*BRCA1* variant	1 [Reference]	1 [Reference]	21.42 (3.86)
**Platinum exposed patients (all stages)**			
*BRCA2* variant	0.60 (0.38-0.94)	0.59 (0.38-0.93)	32.26 (7.62)
*BRCA1* variant	1 [Reference]	1 [Reference]	23.36 (2.89)
**Nonplatinum exposed patients (all stages)**			
*BRCA2* variant	0.65 (0.34-1.22)	0.44 (0.21-0.92)	24.84 (3.52)
*BRCA1* variant	1 [Reference]	1 [Reference]	21.42 (5.39)

Abbreviations: HR, hazard ratio; OS, overall survival.

**Figure.**
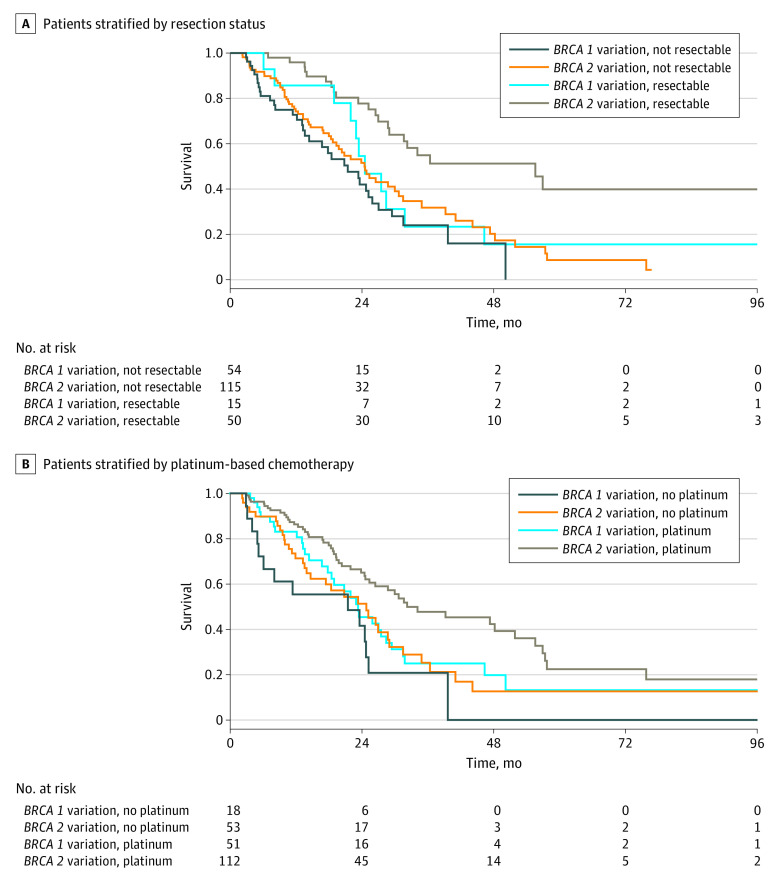
Kaplan-Meier Curves for Overall Survival in Patients With Pancreatic Cancer and *BRCA1* vs *BRCA2* Variants

## Discussion

In a large cohort of patients with germline *BRCA* PVs and pancreatic cancer, *BRCA2* PVs were associated with better outcomes compared with *BRCA1* PVs. This finding appears to be associated with an enhanced benefit from platinum-based therapies and resection in *BRCA2* carriers compared with their *BRCA1*-carrying counterparts. The difference was not statistically significant in the nonresected population.

Biological differences between *BRCA1*-related tumors and *BRCA2*-related tumors have been described. While rates of biallelic loss appear to be similar,^5^ rates of PVs in *TP53* are more common in *BRCA1*-related tumors compared with *BRCA2*-related tumors (88% vs 53%) and PDAC samples with *BRCA2* PVs are more immunogenic than those with *BRCA1* PVs.^6^ This study had limitations including its retrospective nature and the small subgroup of patients with resected PDAC and *BRCA1* PV.

Our findings emphasize the need for a better understanding of possible biological differences between pancreatic cancers that develop in patients with *BRCA1* PVs vs *BRCA2* PVs and consideration of stratification by mutation type in future clinical trials. The association of platinum-based therapies in patients with different BRCA PVs should be evaluated in a separate larger cohort.

## Notes

Supplement.
Data Sharing Statement
