# Supplementary material for: Analysis of BRCA1- and BRCA2-Related Pancreatic Cancer and Survival
Source: JAMA Netw Open. 2023 Nov 27;6(11):e2345013. doi: 10.1001/jamanetworkopen.2023.45013 (PMC10682833; doi:10.1001/jamanetworkopen.2023.45013)
Supplement: Supplement. — Data Sharing Statement [file jamanetwopen-e2345013-s001.pdf]

Data Sharing Statement

Data
Data available: Yes
Data types: Deidentified participant data
How to access data: Kim.ReissBinder@pennmedicine.upenn.edu
When available: With publication

Supporting Documents
Document types: None

Additional Information
Who can access the data: researchers whose proposed use of the data has been approved
Types of analyses: researchers whose proposed use of the data has been approved
Mechanisms of data availability: researchers whose proposed use of the data has been approved and signed data access agreement
